# Functional electrical stimulation-assisted cycle ergometry in the critically ill: protocol for a randomized controlled trial

**DOI:** 10.1186/s13063-019-3745-1

**Published:** 2019-12-16

**Authors:** Petr Waldauf, Jan Gojda, Tomáš Urban, Natália Hrušková, Barbora Blahutová, Marie Hejnová, Kateřina Jiroutková, Michal Fric, Pavel Jánský, Jana Kukulová, Francis Stephens, Kamila Řasová, František Duška

**Affiliations:** 1 Department of Anaesthesiology and Intensive Care Medicine, Charles University, 3rd Faculty of Medicine and KAR FNKV University Hospital, Fac Med 3, Srobarova 50, 10034 Prague, Czech Republic; 2 Department of Internal Medicine II, Charles University, 3rd Faculty of Medicine and FNKV University Hospital, Prague, Czech Republic; 3 Department of Rehabilitation, Charles University, 3rd Faculty of Medicine and FNKV University Hospital, Prague, Czech Republic; 4 College of Life and Environmental Sciences, Sport and Health Sciences, University of Exeter, Exeter, UK

**Keywords:** Early rehabilitation, Critically ill, Intensive care unit, Functional electrical stimulation-assisted cycle ergometry, Mobility, Physical therapy

## Abstract

**Background:**

Intensive care unit (ICU)-acquired weakness is the most important cause of failed functional outcome in survivors of critical care. Most damage occurs during the first week when patients are not cooperative enough with conventional rehabilitation. Functional electrical stimulation-assisted cycle ergometry (FES-CE) applied within 48 h of ICU admission may improve muscle function and long-term outcome.

**Methods:**

An assessor-blinded, pragmatic, single-centre randomized controlled trial will be performed. Adults (*n* = 150) mechanically ventilated for < 48 h from four ICUs who are estimated to need > 7 days of critical care will be randomized (1:1) to receive either standard of care or FES-CE-based intensified rehabilitation, which will continue until ICU discharge. Primary outcome: quality of life measured by 36-Item Short Form Health Survey score at 6 months. Secondary outcomes: functional performance at ICU discharge, muscle mass (vastus ultrasound, N-balance) and function (Medical Research Council score, insulin sensitivity). In a subgroup (*n* = 30) we will assess insulin sensitivity and perform skeletal muscle biopsies to look at mitochondrial function, fibre typing and regulatory protein expression.

**Trial registration:**

ClinicalTrials.gov, NCT02864745. Registered on 12 August 2016.

## Background

Functional disability, a natural consequence of weakness, is a frequent and long-lasting complication in survivors of critical illness [1–3]. Over recent decades, mortality from acute critical illness has decreased with a consequent increasing number of ICU survivors. Understanding the post-ICU morbidity experienced by these survivors has become increasingly important. The greatest burdens that survivors of critical illness face are related to neuromuscular dysfunction and neuropsychological maladjustment [4]. In particular, neuromuscular abnormalities during critical illness are common, with a median prevalence of 57% [1]. In both patients with chronic critical illness and survivors of severe critical illness, neuromuscular weakness may be substantial and persistent [5], resulting in important decrements in physical function and quality of life for years after discharge [1, 2].

In the past, routine features of general care provided in the ICU included liberal use of sedation and immobilization of the patient, which were thought to be necessary for facilitating interventions to normalize physiological function by artificial means. Over the last decade, there has been a paradigm shift away from this approach towards a more conservative treatment philosophy for patients in the ICU [4, 6, 7]. This paradigm shift is consistent with the observation that long-term physical problems in survivors of critical illness, particularly those with respiratory failure, may result from the protracted ICU stay and period of immobilization during which the patient is receiving organ support that is essential for survival [2, 4]. In line with this, a daily interruption of sedation policy has been widely adopted and proven to be beneficial [8] and early mobilization culture is spreading quickly across ICUs [9–13]. Indeed, these strategies, together with early physical therapy [9, 11, 12, 14–20], are the only safe [12, 20–22] and effective interventions in the prevention of long-term neuromuscular disability in survivors of intensive care. It should be stressed that in these studies early rehabilitation is defined as starting between days 2 and 5 of the ICU stay [9, 11, 12, 14–19] or as an activity beginning before ICU discharge [20].

*Standard “early” rehabilitation cannot be started early enough, and FES-CE may be a solution to this dilemma.* The first week in the ICU is critical as muscle mass and function is lost quickly. Immobility-associated muscle loss is evident as early as within 18–48 h of onset of acute critical illness or severe injury [23, 24] and is greatest during the first 2–3 weeks of critical illness [25, 26]. Up to 40% loss of muscle strength can occur within the first week of immobilization, with a daily rate of strength loss between 1.0 and 5.5% [27]. A 10–14% decrease in cross-sectional measurements of the rectus femoris muscle has been observed within the first week of the ICU stay [26]. Conventional rehabilitation during the first few days in the ICU is indeed limited in patients who are sedated and mechanically ventilated, and typically consists of passive limb movements, with or without the use of stretch reflex [16, 20]. Schweickert et al. [16] provided the earliest (within 48 h of intubation) and largest (26 ± 14 min a day for patients on mechanical ventilation) dose of rehabilitation and reported improvements of physical function at hospital discharge, but no measurements beyond. Active rehabilitation is delayed until the neurological condition of the patient improves enough to facilitate participation. In the sickest patients, who are at particular risk of developing ICU-acquired weakness (ICUAW), sedation and immobility may be prolonged well beyond the first week, when established damage to the muscle has already occurred.

There are several ways to deliver more effective physical exercise therapy to patients who are sedated and mechanically ventilated. For example, physical exercise can be delivered effectively and safely by passive supine cycling on a bicycle ergometer [15, 18, 28–30]. More recently, electrical neuromuscular stimulation (NMES) has been developed to mimic active exercise in patients who lack voluntary muscle activity [31–39]. During NMES, cutaneous electrodes placed over specific muscle groups electrically trigger muscle contractions. In order to achieve maximum efficacy, passive cycling and NMES can be delivered simultaneously and synchronized to produce a coordinated pattern of movements. The technique is called FES-CE (functional electrical stimulation-assisted cycle ergometry). There is a large body of experience with these methods in the rehabilitation of patients with stroke and spinal cord injuries (reviewed in [40]). The method is effective in preventing the loss of muscle mass [41] and has been shown to improve anabolic resistance and insulin sensitivity in quadriplegic patients [42, 43].

The only study of FES-CE in critical illness is the pilot trial by Parry et al. [44], where the feasibility and safety of FES-CE was demonstrated in a small cohort of critically ill patients (eight patients received the FES-CE intervention, versus eight controls). Patients in the intervention group showed significant improvements in the Physical Function in Intensive Care Test and a faster recovery of functional milestones (e.g. time to stand from lying, walking on the spot). However, the mechanism by which this occurred is unknown. There are no data on the effect of FES-CE on long-term functional outcome in ICU survivors. In healthy volunteers [45] and patients with spinal cord injury [46], unloaded FES-CE can increase whole-body oxygen consumption. It is unknown whether these effects, including improving insulin sensitivity and protein metabolism [47], can also be achieved in critically ill patients.

### Rationale

#### Mechanisms of muscle wasting and ICUAW

Pathophysiology of ICUAW is complex and multifactorial (reviewed in [4]), and there is a growing body of evidence suggesting the role of sarcopenia and metabolic derangement of skeletal muscle.

Firstly, *insulin resistance* is a well-known comorbidity in critical illness [48], contributing to and aggravating complications such as severe infections, organ dysfunction and death, and has also been implicated in the ICU-acquired weakness. Two main consequences of insulin resistance are hyperglycaemia and “anabolic resistance”. It has been observed that the provision of protein and energy to support the enhanced hypermetabolic demands of ICU patients is unable to prevent the rapid loss of muscle mass [49]. Indeed, skeletal muscle insulin resistance is the likely reason why nutritional support further exacerbates hyperglycaemia. Insulin therapy is often used in ICU patients to try and combat this, but it appears to be ineffective in ICU-acquired weakness and its safety in the ICU setting has been questioned [50]. Physical activity is an attractive alternative intervention target as it has profound effects on substrate metabolism in contracting skeletal muscle, with a single bout of muscle contraction known to increase muscle glucose uptake several fold and sensitize the muscle to insulin and the anabolic effects of amino acids for up to 24 h, including in individuals where insulin and anabolic resistance is evident [51]. It is not known whether intensified rehabilitation can improve the insulin effect on glucose uptake and whether it influences the stimulatory effect of insulin and amino acids on muscle protein synthesis.

Secondly, *mitochondrial dysfunction* in skeletal muscle may play a role in the development of ICUAW. Mitochondrial depletion and dysfunction of mitochondrial respiratory complexes I and IV has been demonstrated in acute severe sepsis in association with multiorgan failure and death [52], and early activation of mitochondrial biogenesis predicted survival [53]. Our group has recently demonstrated in two pilot studies [54, 55] that, compared to healthy controls, there is a 50% reduction of mitochondrial functional capacity in skeletal muscle in the patients with protracted critical illness and ICUAW. This is accompanied by a significant relative increase in the abundance and functional capacity of respiratory complex II, which delivers electrons to the respiratory chain from fatty acid oxidation [54]. Weber-Carstens et al. [48] demonstrated that insulin fails to activate GLUT-4 translocation to cellular membranes in patients with ICUAW, causing skeletal muscle “intracellular glucose starvation” and a failure of AMP-activated protein kinase to respond to the impairment of ATP production. Most notably, in five subjects, these abnormalities were alleviated by NMES. In light of this, the relative increase of complex II capacity observed in our pilot study may represent a functional adaptation of muscle to the increased reliance on fatty acid oxidation. It is not known whether the severity of mitochondrial functional alteration reflects the degree of insulin resistance and the severity of muscle weakness, and whether the delivery of very early FES-CE has a potential to influence these changes.

In the light of this, we hypothesize the following:

- H_1_: As most of the damage to the structure and function of skeletal muscle occurs during the first week, intensified goal-directed rehabilitation, which includes FES-CE and starts within 48 h after ICU admission, improves the functional outcome of ICU survivors at 6 months when compared to the standard of care.
- H_2_: The intervention, as compared to standard of care, shall preserve muscle mass and improve muscle power at ICU discharge.
- H_3_: The intervention, as compared to standard of care, shall increase insulin-mediated whole-body oxidative glucose disposal and mitochondrial functional indices.

### Objectives

1. To investigate, in a pragmatic, prospective, randomized, controlled, assessor-blinded trial, the effects of very early intensive rehabilitation using a goal-directed protocol that includes FES-CE in mechanically ventilated ICU patients predicted to need a protracted ICU stay
2. To perform more detailed metabolic studies, including serial muscle biopsies and using euglycaemic hyperinsulinaemic clamps, in a nested subgroup. Insulin sensitivity in the whole study population will be compared by glucose control and consumption of intravenous insulin required to control blood glucose

#### Primary outcome

The primary outcome is the physical component of the SF-36 quality of life questionnaire measured in ICU survivors at 6 months. Based on the study by Kayambu et al. [12], where this measure was 60 ± 29 points in the control group, our study is powered to detect a change by 15 points or more, which is within the limits determined as clinically important for patients with COPD, asthma and myocardial infarction [56]. The SF-36 has been validated in the Czech Republic and endorsed by the Institution for Health Information and Statistics (https://www.uzis.cz/en/node/8159).

#### Secondary outcomes

- Four-item Physical Fitness in Intensive Care Test (time frame: at 28 days or discharge from the ICU, whichever occurs earlier) as the functional outcome at ICU D/C
- Muscle mass measured by rectus muscle cross-sectional area on B-mode ultrasound (time frame: at 7-day intervals up to day 28 or discharge from the ICU, whichever occurs earlier)
- Nitrogen balance measured in grams per metre-squared of body surface area (time frame: at 7-day intervals up to day 28 day or discharge from the ICU, whichever occurs earlier) and the cumulative the difference between nitrogen intake and output
- Muscle power as per the Medical Research Council (MRC) score (time frame: at 7-day intervals up to day 28 or discharge from the ICU, whichever occurs earlier)
- Number of ventilator-free days (time frame: at 28 days); that is, number of days, out of 28 days after admission, that the patient has NOT been supported by mechanical ventilation
- Number of rehabilitation interruptions due to physiological deterioration (time frame: at 28 days or discharge from the ICU, whichever occurs earlier)
- Number of episodes of elevated intracranial pressure (time frame: at 28 days or discharge from the ICU, whichever occurs earlier)
- Number of dialysis interruptions (time frame: at 28 days or discharge from the ICU, whichever occurs earlier)
- Length of ICU stay in days (time frame: at 6 months)

### Study population

One hundred and fifty participants meeting the eligibility criteria will be recruited in four ICUs at FNKV University Hospital.

Inclusion criteria: age ≥ 18 years; mechanical ventilation, or imminent need of it at presentation; predicted ICU length of stay ≥ 7 days.

Exclusion criteria: known primary systemic neuromuscular disease or spinal cord lesion at admission; severe lower limb injury or amputation; bedridden premorbid state (Charleston Comorbidity Score > 4); approaching imminent death or withdrawal of medical treatment within 24 h; pregnancy; presence of external fixator or superficial metallic implants in lower limbs; open wounds or skin abrasions at electrode application points; presence of pacemaker, implanted defibrillator, or other implanted electronic medical device; predicted as unable to receive first rehabilitation session within 72 h of admission or transferred from another ICU after more than 24 h of mechanical ventilation; presence of other condition preventing the use of FES-CE or considered unsuitable for the study by a responsible medical team; prior participation in another functional outcome-based intervention research study.

With the exception that we do not limit the study population with sepsis, we have intentionally chosen similar criteria to the only study underway on FES-CE in ICU patients, which is primarily focused on muscle structure and function [57].

### Interventions

**Fig. 1:**
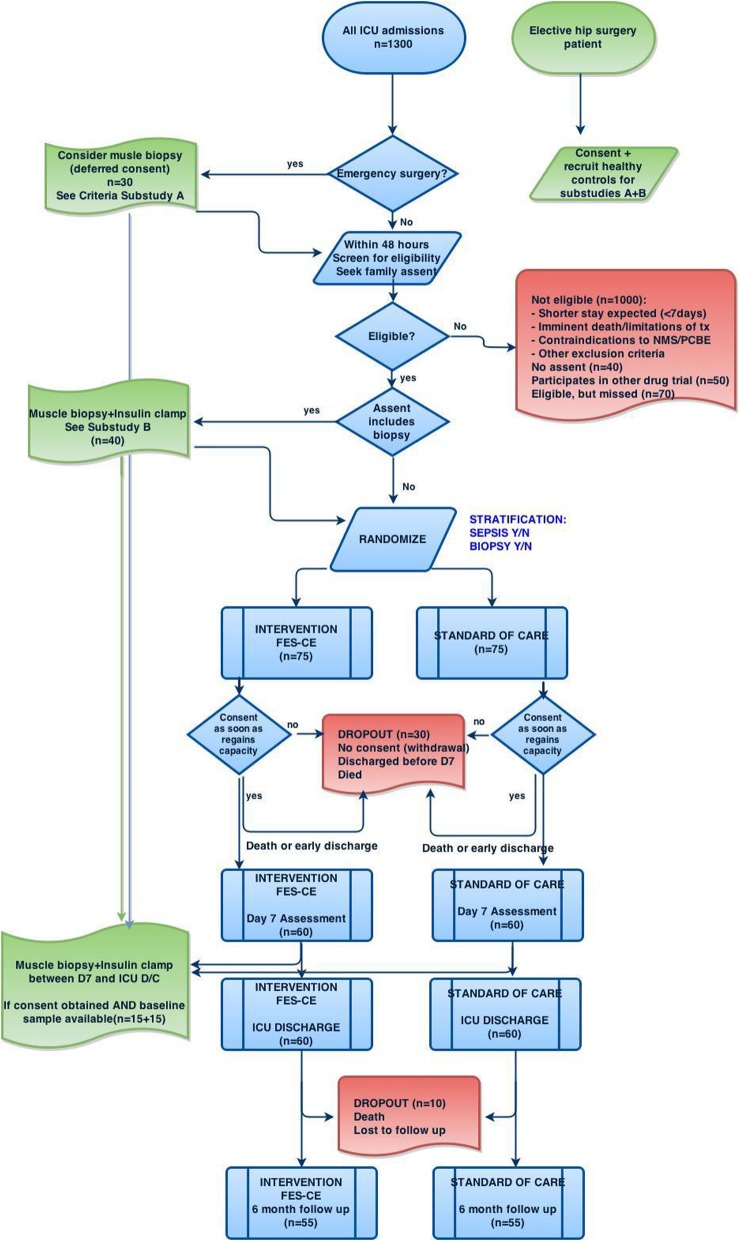
Planned flowchart of patients enrolled into the trial. D7 day 7, D/C discharge, FES-CE functional electrical stimulation-assisted cycle ergometry, ICU intensive care unit, tx treatment, NMS neuromuscular stimulation, PCBE passive cycling-based exercise

**Fig. 2:**
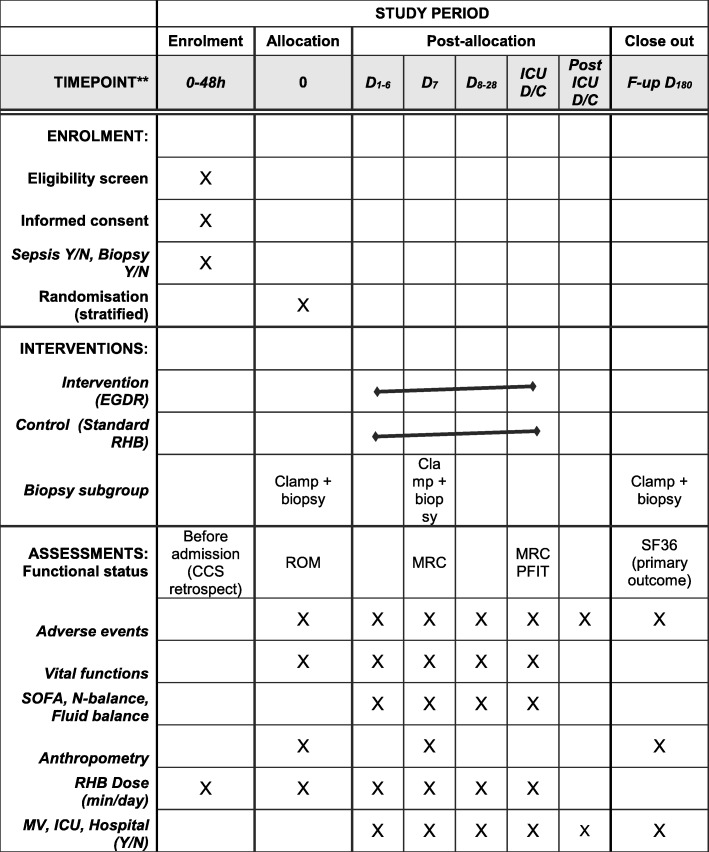
Standard Protocol Items: Recommendations for Interventional Trials (SPIRIT) figure. D day, D/C discharge, EGDR early goal-directed rehabilitation, F-up follow-up, ICU intensive care unit, MRC Medical Research Council, MV mechanical ventilation, RHB rehabilitation, SF-36 Short Form 36, SOFA Sequential Organ Failure Assessment, CCS Charlson comorbidity score, ROM range of motion, PFIT physical function test for use in the intensive care unit

The flow of participants throughout the trial is shown in Fig. 1 and the study procedures in Fig. 2. As soon as informed consent has been obtained, and prior to randomization, baseline testing including anthropometric examination will be performed. In addition, in patients with specific consent, a muscle biopsy will be obtained and hyperinsulinaemic clamp will be performed on the first morning (8.00–11.00 a.m.) and prior to the start of enteral nutrition.

#### Standard care group

Both groups will receive usual best medical and nursing care in the ICU, which includes daily sedation holds when applicable and delirium 12-hourly monitoring (by CAM-ICU scale [58]) and management as usual in the routine practice. Respiratory physiotherapy will also be delivered without alterations. The routine standard care arm will undergo mobilization/rehabilitation delivered by personnel not involved in the study in a usual, routine way. Details of physiotherapy treatment will be recorded but not protocolled in the standard care arm.

#### Intervention group

**Fig. 3:**
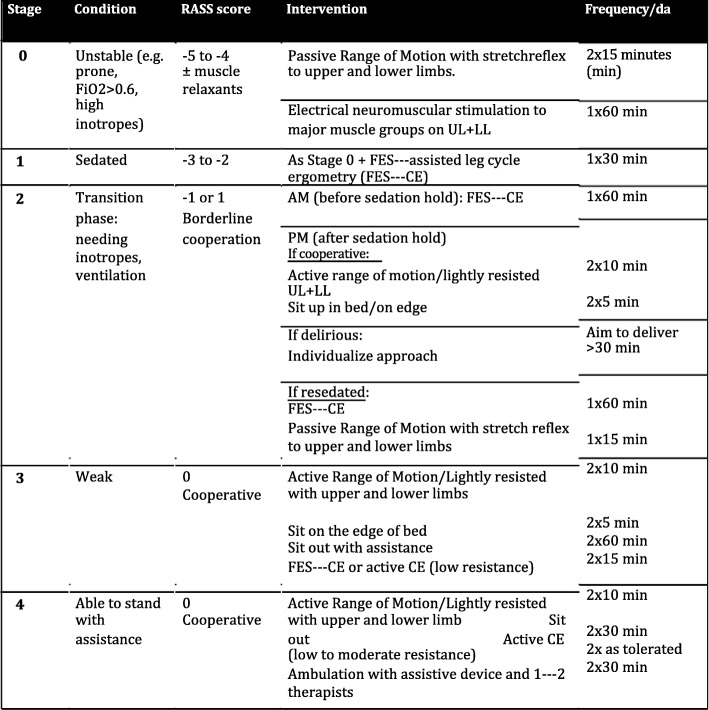
Protocol of intensified goal-directed rehabilitation. FES-CE functional electrical stimulation-assisted cycle ergometry, FIO_2_ fraction of inspired oxygen, LL lower limb, RASS Richmond Agitation and Sedation Scale, UL upper limb

In the intervention arm, early goal-directed rehabilitation is protocolled according to the patients’ condition and degree of cooperation (Fig. 3), and there will be pre-defined safety criteria, which are in accordance with current recommendations for active rehabilitation of critically ill ventilated adults [13]. Whilst the safety criteria are binding for the study physiotherapist, the rehabilitation protocol is not and the delivery of physical exercise can be altered according to the actual patient’s condition. However, any alteration and the reason for it will be recorded. The intervention will start as soon as possible and always within 72 h of ICU admission, continuing until ICU discharge. Supine cycling will be delivered as per protocol on a supine cycle ergometer attached to a neuromuscular stimulator. Surface electrodes will be applied to the gluteal, hamstring and quadriceps muscles on both legs. The intensity of muscle stimulation will be delivered at a level able to cause visible contractions (confirmed by palpation if uncertain) in all muscle groups without causing undue pain or discomfort to the participant, according to a regime specified by Parry et al. [44]. Once the patient is more alert, and able to participate, they will be provided with standardized encouragement to engage in therapy. To increase the intervention workload, resistance will be increased incrementally and cycling cadence. If a participant is readmitted to intensive care, the intervention will be re-initiated. The intervention continues until day 28 or ICU discharge, whichever occurs earlier.

## Methods

### Enrolment and randomization

All patients admitted to participating ICUs are screened daily by research nurses and all eligible patients or their representatives are approached by investigators as soon as possible, but always within 72 h of admission. Participants for whom informed consent was obtained will be randomly assigned (1:1) to receive either standard care or the intervention using offsite independent randomization protocols (www.randomization.com) embedded in the electronic case report form. Randomization will be stratified according to the presence or absence of sepsis and the availability of a biopsy at baseline. There is no restriction (blocking) during randomization.

Both the study team and clinical personnel will be made aware of subject treatment allocation. The outcome assessor is not involved in patient care and remains blinded to treatment allocations.

### Clinical data retrieval and handling

The ICUs are paperless and fully computerized, so vital functions and other physiological parameters are monitored and data are routinely stored in secure hospital data bases via a protected dedicated network (MetaVision; IMD Soft Inc.). This includes data about nutritional intake and urinary output. On top of this, research nurses will input data into an electronic, secure, customized online case report form database (eCRF; accessible at https://195.113.79.251:9090/apex/f?p=103:101:14992036032980). Data protection and encryption is in accordance with the EU’s General Data Protection Regulations as well as Czech data protection laws. The data will be audited by on a regular basis, but at least after each 10 patients are enrolled, by independent study monitor. After the database is locked upon study completion, patients’ data will be de-identified and available in full in a public database.

Urine samples will be collected daily, surfaced with toluene and stored in a deep-freeze facility for later determination of nitrogen content and 3-methyl histidine levels (to calculate the muscle catabolism rate and nitrogen balance). In addition, all study patients will undergo assessment by a study physiotherapist, which includes a measurement of rectus muscle cross-sectional area on both legs and, whenever the patient regains consciousness, also muscle power by MRC score (standardized testing of muscle power (0–5) for 12 muscle groups on all four limbs, giving a score of 0–60 (60 suggesting normal muscle power)). Blood will be taken, and plasma separated and frozen at − 80 °C for later analysis of cytokines and hormone levels. This assessment will be repeated at day 7 intervals and at ICU discharge. At ICU discharge, the patients and relatives will be asked to provide contact details for follow-up. After 6 months, the patient or family will be contacted for structured interview as required for the SF-36 questionnaire, and collected using the RAND methodology (www.rand.org). Whilst participants and the intervention physiotherapist cannot be blinded to group allocation, research staff assessing the outcome will be from a separate clinical department (JG, BB, MH) and thus will remain blinded to treatment allocation. Outcome assessors are familiar with the SF-36, which is in routine use for other trials, and received SF-36 re-training at induction to this trial. Strategies to improve adherence to intervention mainly include the 24/7 availability of one of the team of five research nurses as well as one full-time physiotherapist equivalent only reserved for study interventions, with extra budgeting to cover physiotherapy sessions in the intervention group during the weekend. The time of physiotherapy sessions will be recorded by the physiotherapist and randomly checked by a hidden independent assessor (bedside ICU nurse receiving specific instructions). The primary outcome has been chosen also with respect to the fact that it can be collected over a structured telephone interview, thereby minimizing missing data.

### Complementary studies: insulin resistance and mitochondrial function

These studies will be performed in addition to other study procedures in a nested subgroup of patients, who give specific consent. The first measurement will be performed at baseline prior to randomization, ideally the next morning after admission. Second measurement will be performed on day 7 of ICU stay, i.e. after at least 5 days of intervention.

#### Muscle biopsy

Muscle biopsy will be performed from the vastus lateralis muscle using the Bergstrom needle biopsy technique. The sample will be separated into three parts (50–100 mg each). One part will be immediately frozen in liquid nitrogen for analysis of the protein/DNA ratio and for protein expression studies. The second part will be frozen in liquid nitrogen-cooled isopentone for muscle fibre typing and immunohistochemistry analysis. The third part put will be placed in BIOPS media on ice for the preparation of homogenates and measurement of citrate synthase activity, spectrophotometric analysis of the activity of respiratory complexes I–IV [52] and western blot analysis of respiratory complexes (as described in [55]). In the fresh muscle homogenates, we will use high-resolution respirometry (Oxygraph; Oroboros, Austria) to determine the function of individual respiratory complexes in the cytosolic context and measure basic functional metabolic indices by a method we have recently developed and calibrated against isolated mitochondria [59]. We will specifically look at the degree of mitochondrial uncoupling, the respiratory chain capacity and the function of individual complexes, including glycerol-3-phosphate shuttle. From the satellite cells we will prepare a culture of myotubes, which will serve as an in vitro model of skeletal muscle [60] and specifically measure the in vitro ability of myotubes to oxidize fatty acids by extracellular flux analysis (Seahorse Biosciences). Frozen muscle samples will be stored at − 80 °C for analysis of the DNA/protein ratio, mRNA and proteins involved in the regulation of proteolysis, substrate oxidation and anabolic pathways of skeletal muscle (MuRF, FOXO, atrogins) as well as immunohistochemistry and typing of muscle fibres. In order to determine which changes are caused by critical illness itself, we will also obtain control samples (*n* = 15) from age, sex and BMI-matched metabolically healthy volunteers undergoing elective hip surgery at the Department of Orthopaedic Surgery. In addition, we will look at the change of these indices after 7 days of critical illness and the influence of the intervention versus standard of care. We will look at correlation of these parameters with muscle power (i.e. compare the bioenergetics profile of skeletal muscle in those who develop ICUAW and in those who do not) and insulin resistance.

*Insulin sensitivity* and substrate oxidation will be measured after overnight fasting by hyperinsulinaemic euglycemic clamp (as described in [61]). We will compare the effect of intervention on insulin-mediated glucose disposal.

### Statistical analyses

#### Sample-size calculation

In studies of critical illness outcome at 6 months using SF-36 scores, the standard deviation varied between 10 and 30 points. In order to have 80% power to detect a 15-point difference in SF-36 scores between control and intervention at the level of significance *p* < 0.05 in the population with a mean of 60 and SD of 30 [12], we would need 108 subjects (54 in each arm). In order to allow for deaths and dropouts, we plan to randomize 150 subjects.

#### Data analysis plan

The primary outcome and all secondary outcomes will be compared between the intervention and standard of care groups in an intention-to-treat population, with all tests two-sided and with the level of significance set at 5%, after the primary outcome has been collected in the last subject. There is no plan for any interim analysis. We will perform exploratory analyses in pre-specified subgroups of patients stratified according to APACHE II, and the length of intervention. We will also perform unadjusted analysis of odds ratio of being functionally independent (defined as ability to walk, use a telephone, self-care, use the toilet and groom) at 6 months after ICU admission in patients in the intervention and standard of care groups. We will perform adjustments on the disease severity (APACHE II score), admission diagnosis, baseline functional status and age. Missing data for primary outcome will be dealt by reporting both worst-case and per-protocol results; no imputation will be used.

### Ethical considerations

This trial involves a two-tier consent process: first to the rehabilitation intervention and then additionally to the insulin clamp and muscle biopsies in a nested subgroup within the primary trial. All patients meeting the aforementioned criteria will be invited to participate and asked to provide written informed consent. It is expected that most screened patients will lack the capacity to provide informed consent. In this situation, the deferred consent policy will be applied: patient’s next of kin (NOK) will be approached, and be given verbal and written information explaining the nature of the study given information leaflet and asked to provide assent. Discussion with the family will help inform the treating medical team regarding a best interest decision for assent to be recruited into the study. An option will be given to participate in the trial, but not to undergo insulin clamps and muscle biopsies. In a subgroup of patients when the family is unavailable within the first 48 h, an independent physician will be asked to review inclusion and exclusion criteria and weight benefits and risks of participation in the trial – all patients enrolled based on independent physician assent will proceed without insulin clamps and muscle biopsies. Participants themselves will be asked to provide ongoing consent as soon as they regain capacity. Again, they will be offered the option to continue participating in the trial without insulin clamps and/or muscle biopsies, if they so wish. Details of all participants who refuse consent for muscle biopsy/insulin clams will be recorded. All serious adverse events that are suspected as being related to study interventions will be reposted to the Research Ethics Board and regulatory authorities as per local legislation. Other adverse events deemed to be related or possibly related to treatment intervention will be discussed at regular monthly meetings of the study team with the decision on further action, as there is no formal steering committee for this trial. The final decision-making and reporting responsibility is with the principal investigator (FD). All adverse events will be recorded in the eCRF. All protocol amendments, should they be required, will be subjected to a priori approval by the REB. Once implemented, protocol amendments will be reported to the sponsor and registration body (www.clinicaltrials.gov).

#### Replication of key aspects of trial methods and conduct

The trial is designed to be fully reproducible in an ICU setting in larger, but not necessarily teaching or academic, hospitals, where the FES-CE equipment and trained physiotherapists are available 7 days a week.

The sponsor of the study is a state-governed grant agency that has not had nor will have any role in study design; collection, management, analysis and interpretation of data; writing of the report; or the decision to submit the report for publication.

## Dissemination of results

We will submit the main results of the trial in an open-access peer-reviewed journal within 6 months after the 150th subject has completed the 6-month follow-up visit, which is expected to happen in Q2 of 2020. We will make fully de-identified record-level raw data available in a public database Additional file 2.

## Trial status

This trial is recruiting (recruitment began November 2016, expected finish November 2019) (first patient recruited 4 October 2016, expected end of study 1 July 2020), protocol version 2.0 as of January 2018. For the full WHO Trial Registration Data Set, see Additional file 1.

## Supplementary information

**Additional file 1.** WHO Trial Registration data set.

**Additional file 2.** SPIRIT 2013 Checklist: Recommended items to address in a clinical trial protocol and related documents.

## Data Availability

All cleaned de-identified raw data will be made available in an open online database (https://data.mendeley.com/datasets) within 6 months of publication of the main results of the trial.

## Abbreviations

D/C: Discharge
FES-CE: Functional electrical stimulation-assisted cycle ergometry
ICU: Intensive care unit
MRC: Medical Research Council
SF-36: 36-Item Short Form Health Survey
