# Supplementary material for: Functional electrical stimulation-assisted cycle ergometry in the critically ill: protocol for a randomized controlled trial
Source: Trials. 2019 Dec 16;20:724. doi: 10.1186/s13063-019-3745-1 (PMC6915865; doi:10.1186/s13063-019-3745-1)
Supplement: Supplementary file 1 — Additional file 1. WHO Trial Registration data set. [file 13063_2019_3745_MOESM1_ESM.docx]

1. **Primary Registry and Trial Identifying Number**
   Clinical Trials ([www.clinicaltrials.gov](http://www.clinicaltrials.gov)) NCT02864745
2. **Date of Registration in Primary Registry**
   August 12, 2016
3. **Secondary Identifying Numbers**
   - Identifiers assigned by the sponsor: AZV-16-28663A
   - Other trial registration numbers issued by other Registries (both Primary and Partner Registries in the WHO Registry Network, and other registries): NA
   - Identifiers issued by ethics committee/ institutional review boards: EK-VP/27/0/2015

1. **Source(s) of Monetary or Material Support**
   Monetary and material support for the trial has been provided exclusively by Agentura pro Zdravotnicky Vyzkum (AZV, Agency for Health Research, which is Czech government’s grant agency)
2. **Primary Sponsor**
   Charles University, 3^rd^ Medical Faculty, Prague, Czech Republic
3. **Secondary Sponsor(s)**
   FNKV University Hospital in Prague (Fakultni Nemocnice Kralovske Vinohrady)
4. **Contact for Public Queries**.

Dept. of Anaesthesia and Intensive Care, Charles University, Fac Med 3 and FNKV University Hospital

Postal: Srobarova 50. PSC13000 Prague, Czech Republic

Telephone: +420267262451

E-mail: karsec@fnkv.cz

1. **Contact for Scientific Queries**
   Dr Frantisek Duska, Principal Investigator

Dept. of Anaesthesia and Intensive Care, Charles University, Fac Med 3 and FNKV University Hospital

Postal: Srobarova 50. PSC13000 Prague, Czech Republic

Telephone: +420267262461

E-mail: [frantisek.duska@lf3.cuni.cz](mailto:frantisek.duska@lf3.cuni.cz)

1. **Public Title**
   Electrical muscle stimulation to improve recovery after critical illness

**Scientific Title**
Functional Electrical Stimulation‐Assisted Cycle Ergometry in Critically Ill. Protocol of Randomised Controlled Trial

Abbreviated title: Early Mobilization and Intensive Rehabilitation in the critically ill

Acronym EMIR

1. **Countries of Recruitment**
   Czech Republic
2. **Health Condition(s) or Problem(s) Studied**
   Primary health condition(s) or problem(s) studied: Functional disability after surviving critical illness
3. **Intervention(s)**
   For each arm of the trial record a brief intervention name plus an intervention description.

   Intervention Name: Functional electrical stimulation-asisted cycle ergometry-based early rehabilitation

Control Arm: Standard-of-care Rehabilitation

1. **Key Inclusion and Exclusion Criteria**
   **Inclusion Criteria**: (1) ≥18 years; (2) mechanical ventilation, or imminent need of it at presentation; (3) predicted ICU length of stay ≥7 days;

**Exclusion Criteria**: (1) known primary systemic neuromuscular disease or spinal cord lesion at admission. (2) severe lower limb injury or amputation; (3) bedridden premorbid state (Charleston Comorbidity Score >4) (4) approaching imminent death or withdrawal of medical treatment within 24 h; (5) pregnancy; (6) presence of external fixator or superficial metallic implants in lower limb; (7) open wounds or skin abrasions at electrode application points; (8) presence of pacemaker, implanted defibrillator or other implanted electronic medical device; (9) predicted as unable to receive first rehabilitation session within 72 hours of admission or transferred from another ICU after more than 24 hours of mechanical ventilation; (10) Presence of other condition preventing the use of FESCE or considered unsuitable for the study by a responsible medical team; (11) prior participating in another functional outcome-based intervention research study.

1. **Study Type**
   Study type consists of:
   - Type of study: interventional
   - Study design including:
     - randomized
     - outcome assessor blinded
     - parallel
   - Phase II.
2. **Date of First Enrollment**
   4st October 2016
3. **Sample Size**
   of:
   - Number of participants that the trial plans to enrol in total = 150
   - Number of participants that the trial has enrolled = 133
4. **Recruitment Status**
   Recruitment status of this trial: **Recruiting**
5. **Primary Outcome**
   - The name of the outcome (do not use abbreviations): Physical component of SF-36 quality of life questionnaire measured in ICU survivors
   - The metric or method of measurement used (be as specific as possible): Calculate physical component score of SF-36 questionnaire and compare between intervention and contro group
   - The timepoint(s) of primary interest: 6 month after randomisation

1. **Key Secondary Outcomes**

- 4‐item Physical Fitness in Intensive Care test [Time Frame: at 28 days or discharge from ICU whichever occurs earlier ] as functional outcome at ICU d/c
- Muscle mass measured by rectus m. cross sectional area on B-mode ultrasound [Time Frame: at 7 day intervals up to 28th day or discharge from ICU, whichever occurs earlier]
- Nitrogen balance measured in g/m2 of body surface area [Time Frame: at 7 day intervals up to 28th day or discharge from ICU, whichever occurs earlier] and The cumulative the difference between nitrogen intake and output
- Muscle power per Medical Research Council (MRC) score [Time Frame: at 7 day intervals up to 28th day or discharge from ICU, whichever occurs earlier ]
- Number of ventilator-free days [Time Frame: at 28 day ], i.e. number of days, out of 28 after admission, patient has NOT been supported by mechanical ventilation
- Number of rehabilitation interruptions due to physiological deterioration [Time Frame: at 28 days or discharge from ICU whichever occurs earlier ]
- Number of episodes of elevated intracranial pressure [ Time Frame: at 28 days or discharge from ICU whichever occurs earlier]

1. **Ethics Review**
   The ethics review process information of the trial record in the primary register database. It consists of:
   - Status: Approved
   - Date of approval: 24^th^ June 2015

Name and contact details of Ethics committee(s): prof. Jan Pachl, Chairman, FNKV Univ Hospital Ethical Committee, Postal: Srobarova 50. PSC13000 Prague, Czech Republic.

Telephone: +420267261111

E-mail: eticka.komise@fnkv.cz

1. **Completion date**
   Date of study completion: Not applicable (expected 2020)
2. **Summary Results**: Not applicable.
3. **IPD sharing statement**
   - Plan to share IPD: Yes
   - Plan description: Remove all patients identifiers, move timeframe of events to prevent untoward de-identification and upload record-level data to a public database. Embargo access for 6 months from the date the main paper is published.
